# *PwRn1*, a novel *Ty3/gypsy*-like retrotransposon of *Paragonimus westermani*: molecular characters and its differentially preserved mobile potential according to host chromosomal polyploidy

**DOI:** 10.1186/1471-2164-9-482

**Published:** 2008-10-14

**Authors:** Young-An Bae, Jong-Sook Ahn, Seon-Hee Kim, Mun-Gan Rhyu, Yoon Kong, Seung-Yull Cho

**Affiliations:** 1 Department of Molecular Parasitology and Samsung Biomedical Research Institute, Sungkyunkwan University School of Medicine, Suwon, Gyeonggi-do 440-746, Korea; 2 Department of Microbiology, College of Medicine, Catholic University of Korea, Seoul 137-701, Korea

## Abstract

**Background:**

Retrotransposons have been known to involve in the remodeling and evolution of host genome. These reverse transcribing elements, which show a complex evolutionary pathway with diverse intermediate forms, have been comprehensively analyzed from a wide range of host genomes, while the information remains limited to only a few species in the phylum Platyhelminthes.

**Results:**

A LTR retrotransposon and its homologs with a strong phylogenetic affinity toward *CsRn1 *of *Clonorchis sinensis *were isolated from a trematode parasite *Paragonimus westermani *via a degenerate PCR method and from an insect species *Anopheles gambiae *by *in silico *analysis of the whole mosquito genome, respectively. These elements, designated *PwRn1 *and *AgCR-1 *– *AgCR-14 *conserved unique features including a t-RNA^Trp ^primer binding site and the unusual CHCC signature of Gag proteins. Their flanking LTRs displayed >97% nucleotide identities and thus, these elements were likely to have expanded recently in the trematode and insect genomes. They evolved heterogeneous expression strategies: a single fused ORF, two separate ORFs with an identical reading frame and two ORFs overlapped by -1 frameshifting. Phylogenetic analyses suggested that the elements with the separate ORFs had evolved from an ancestral form(s) with the overlapped ORFs. The mobile potential of *PwRn1 *was likely to be maintained differentially in association with the karyotype of host genomes, as was examined by the presence/absence of intergenomic polymorphism and mRNA transcripts.

**Conclusion:**

Our results on the structural diversity of *CsRn1*-like elements can provide a molecular tool to dissect a more detailed evolutionary episode of LTR retrotransposons. The *PwRn1*-associated genomic polymorphism, which is substantial in diploids, will also be informative in addressing genomic diversification following inter-/intra-specific hybridization in *P. westermani *populations.

## Background

Retrotransposons, which comprise a major portion of eukaryotic genomes, replicate progeny copies into new genomic loci via reverse transcription of an RNA intermediate [1]. As intragenomic parasites, retrotransposons have been known as a potent causative agent involved in various harmful biological processes such as insertional mutagenesis [2] and ectopic recombination [3]. Conversely, cumulative data have evidenced that these elements play significant roles in the formation and maintenance of host chromosomes [4]. The sporadic expansion of retrotransposons can also introduce genomic/phenotypic variants, which lead to speciation in the long evolutionary terms [5,6]. Retrotransposons are subdivided into two large categories, long-terminal-repeat (LTR) and non-LTR retrotransposons, according to overall structures, and the elements with LTR seem to be the most abundant type in invertebrates. The LTR retrotransposons include multiple groups, as have been distinguished by comparing their own gene products [7,8].

In addition to the typical LTR retrotransposons, multiple elements with unusual structural features have recently been isolated from various genomes, such as *CsRn1 *of *Clonorchis sinensis *[9], *Xena *of *Takifugu rubripes *[10], *Gmr1 *of *Gadus morhua *[11] and *Saci-2 *of *Schistosoma mansoni *[12]. The *CsRn1 *and *Saci-2 *elements were found to encode Gag with unique motifs of CHCC and CCCH, respectively, instead of the conventional CCHC. *Gmr1 *produced a Pol protein, in which the functional protein domains lie in an order identical to those of *Ty1/copia *elements (protease [PR]-integrase [IN]-reverse transcriptase [RT]-RNase H [RH]). Retrotransposons of the most ancient *Xena *group had a single open reading frame (ORF) containing a RT domain but lacking any other enzymatic domain. Taken together, these facts have suggested that the category and evolutionary episode of the diverse reverse-transcribing elements are more complex than currently understood.

*Paragonimus westermani *is a hermaphroditic, digenetic trematode that lives as adult in the lungs of carnivorous mammals. This parasite causes pulmonary and cerebral diseases in humans and is one of the most medically important flukes. The natural populations of *P. westermani *in northeast Asian countries have three different levels of polyploidy in their genomic contents, *i*.*e*., di-, tri- and tetrapolyploidy, displaying variations in morphology, allozyme patterns, and nucleotide sequences of ribosomal and mitochondrial DNAs [13]. The *Paragonimus *genome contains retrotransposons, which belong to various retrotransposon groups including the *CsRn1 *clade [14]. *CsRn1*-like retrotransposons have been detected largely in trematodes and insects [9,12,15]. With the unique Gag motif and amino acid conservation, members of the clade showed heterogeneity in their coding profiles. Furthermore, *Boudicca *and *Saci-3 *of *S. mansoni *contain a third ORF resembling envelope protein (ENV) of errantivirus and retrovirus [12,16].

The genomic distribution of *CsRn1*-like elements have been well described in *C. sinensis *and *S. mansoni*, with their unique structural features [9,12,16]. Nevertheless, molecular information on this distinctive clade is highly limited to address their evolutionary episodes. In this study, we isolated a novel *CsRn1*-like LTR retrotransposon in the *P. westermani *genome and analyzed its intra- and inter-genomic distribution patterns among the parasite populations with different karyotes. Differential replication activity of the *Paragonimus *element was examined by detecting the presence or absence of mRNA transcripts and intergenomic polymorphism introduced by the element. The molecular characters of multiple retrotransposons homologous to the element were also analyzed from the genomes of an African malaria mosquito *Anopheles gambiae*, which had recently been released [17], and a fruit fly *Drosophila melanogaster*, in order to gain more detailed information on the invertebrate-specific *CsRn1 *clade. The heterogeneous expression strategies were found to be substantial within the unique clade, and the possible evolutionary relationships between elements with distinctive coding profiles were shown by phylogenetic analyses.

## Results

### Isolation of *CsRn1*-like retrotransposons from *P. westermani *and *A. gambiae*

**Figure 1:**
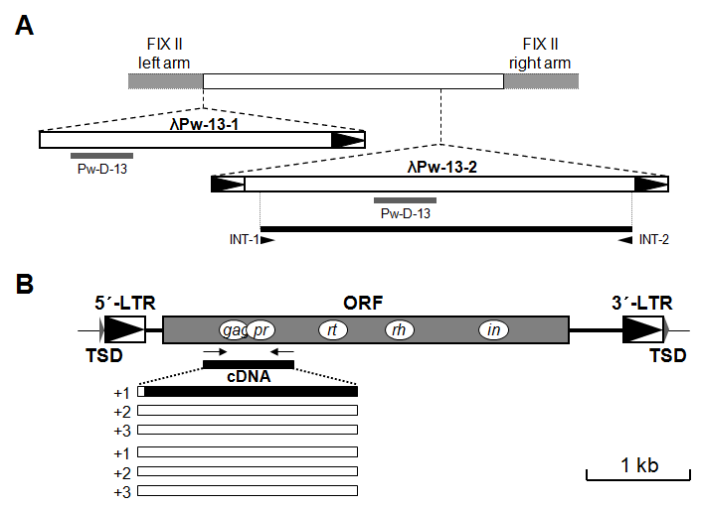
**Isolation and structural characterization of a *CsRn1*-like element from the genome of *Paragonimus westermani***. (A) Schematic representation of an LTR retrotransposon contained in the genomic lambda clones (λPw-13-1 and λPw-13-2), which were homologous to the Pw-D-13 probe. Boxes with a filled arrowhead indicate a direct repeat sequence. The primer positions used in the retrieval of multiple copy sequences are also shown (INT-1, 5'-AGTTGGAAGCCACATTCGCGCTACG-3' and INT-2, 5'-GTGACAACAACCCTTCGATCCTGATG-3'). (B) Overall structure of the *Paragonimus *retrotransposon, named *PwRn1*. Gray box represents an open reading frame (ORF) encoding Gag, protease (PR), reverse transcriptase (RT), RNase H (RH) and integrase (IN). Duplicated target sites of 4 bp are indicated as target site duplication (TSD). The *gag*-*pol *boundary region was further verified by the nucleotide sequences of cDNA clones (see also the legend for Figure 6).

Using the previously described degenerate primers, retrotransposon-related sequences had been isolated in *P. westermani *[14]. These sequences represented various LTR retrotransposons belonging to the multiple clades of *Metavirus *(*Ty3/gypsy *group) including the *CsRn1 *clade, of which members seemed to expand uniquely in lower animal taxa such as Insecta and Trematoda [9,12]. One clone (Pw-D-13 [GenBank:BZ715464]) showing the highest sequence identity with *CsRn1 *was used as a probe in the screening of the genomic DNA library of the parasite. As shown in Figure 1, the full-unit LTR retrotransposon encompassing the probe sequence was determined and named *PwRn1 *(*P*. *westermani Retrotransposon1*) by comparing sequences of two positive clones (λPw-13-1 [GenBank:AY237161] and λPw-13-2 [GenBank:AY237162]). The *PwRn1 *copy in λPw-13-2 was 5,400-bp long and was bound by direct repeats of 4 bp (5'-GGCG-3') known as target site duplication (TSD). The internal sequence was corrupted by several stop codons introduced by indels and/or base substitutions, although its flanking 5'- and 3'-LTRs had an identical size (381 bp) and a high degree of sequence identity (99.7%). The internal coding regions were further retrieved from different *PwRn1 *copies (35 copies [GenBank:EU622539 – EU622573], Avg. divergence = 0.051 ± 0.002) (Figure 1A). The coding profile of *PwRn1 *was predicted with one of these sequences (PwRn1-Int-29 [GenBank:EU622567]), which contained the longest ORF of 3,585 bp encoding a single 1194-aa polypeptide. The corrupted nucleotides in the 5'- and 3'-upstream regions of the ORF were corrected by comparing them with the equivalent regions in a consensus sequence determined from the 35 sequences. The *gag*-*pol *boundary region was further confirmed by sequencing the corresponding cDNAs (Figure 1B).

**Figure 2:**
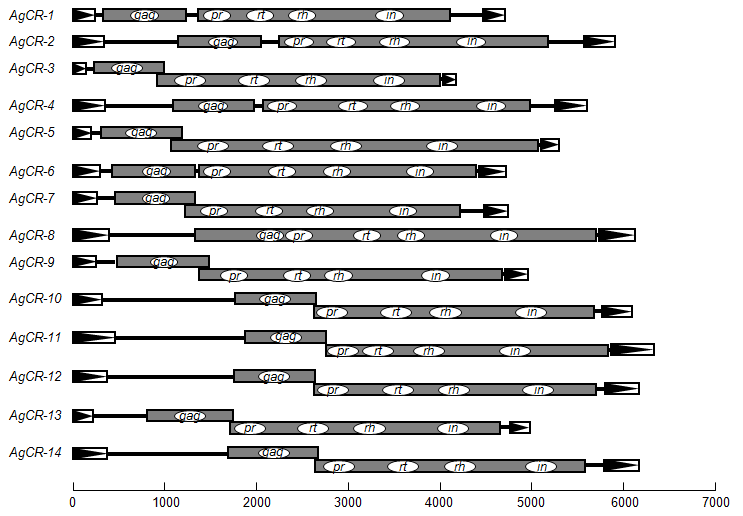
**Schematic diagram showing the overall structures and expression strategies of *AgCR *elements isolated from the genomic sequences of *Anopheles gambiae***. The coding profiles within each of the elements were predicted from a representative copy, as listed in Table 1, of which disrupted bases had been corrected by referencing a consensus sequence of multiple copies.

The genomic sequences of *A. gambiae *in the GenBank database were examined by using the amino acid sequence of *CsRn1 *Gag protein as a query (TBLASTN algorithm). The nucleotide sequences of matching entries were analyzed via a series of BLASTN searches to determine the full-unit retrotransposons encompassing each of the *gag *sequences. The integrity and identity of the elements were verified by detecting a TSD pair at the boundary regions, and by comparing lengths and nucleotide sequences of their LTRs, respectively. These procedures isolated a total of 14 *CsRn1*-like retrotransposons from the mosquito genome, which were designated *AgCR-1 *– *AgCR-14 *(*A*. *gambiae CsRn1*-like Retrotransposon) (Table 1). The complete sequences were used as a BLAST query to retrieve additional copy sequences from the genome. The overall structures and coding profiles were predicted, as described above (Figure 2). One-half of the *AgCR *elements were found to be identical to the *CsRn1*-clade members previously reported [15] (Table 1). *CsRn1*-like elements could not be retrieved from the genomic sequences of *D. melanogaster*, other than the retrotransposon on AE003787 [9], named *DmCR-1 *in this study.

**Table 1:** *CsRn1*-like retrotransposons of *Anopheles gambiae *isolated in this study

Element	Contig No. (nucleotide position)^a^	Nucleotide lengths (bp)				LTR identity (%)	TSD	Note^d^
					
		5'-/3'-LTR	Full unit	ORF1^b^	ORF2^b, c^			
*AgCR1*	AAAB01008980 (3956962–3961632)	264/264	4671	978	2961 (0)	98.1	AAGC	
*AgCR2*	AAAB01008810 (41113–47019)	368/368	5907	978	3168 (0)	100	AACT	GYPSY51AG
*AgCR3*	AAAB01008968 (1135463–1139639)	146/146	4177	756	3090 (-1)	100	AATG	GYPSY50AG
*AgCR4*	AAAB01008984 (9436961–9442636)	356/356	5676	948	3135 (0)	100	AACT	GYPSY70AG
*AgCR5*	AAAB01008831 (1206–6507)	202/202	5302	882	2993 (-1)	100	CGTC	
*AgCR6*	AAAB01008880 (20632–25306)	300/300	4675	888	3087 (0)	99.7	CACC	GYPSY53AG
*AgCR7*	AAAB01008850 (51014–56635)	266/267	5622	876	2856 (-1)	97.3	AAGT	
*AgCR8*	AAAB01008888 (3231118–3237246)	394/394	6126	984	3249 (0)	100	GCAT	GYPSY52AG
*AgCR9*	AAAB01008851 (1315535–1320623)	260/260	5089	915	2964 (-1)	100	GGTT	
*AgCR10*	AAAB01008869 (24283–30418)	319/316	6136	885	2991 (-1)	99.1	ATGT	GYPSY49AG
*AgCR11*	AAAB01008957 (186235–192486)	407/407	6252	909	3081 (-1)	98.5	TATC	
*AgCR12*	AAAB01008964 (2714632–2720796)	370/370	6165	894	3084 (-1)	100	AGTT	GYPSY48AG
*AgCR13*	AAAB01008849 (1694453–1699416)	219/206^e^	4964	933	2946 (-1)	99.5	AGGG^e^	
*AgCR14*	AAAB01008944 (738407–744580)	383/383	6174	984	2970 (-1)	100	ACCC	

^a^Contig sequence containing a representative copy is listed.

^b^The lengths of ORFs were estimated from each of the elements, after correcting the corrupted nucleotide sequences (see text).

^c^Number in parentheses indicates a shift in the reading frame of ORF2 compared to that of ORF1.

^d^Synonymous names, as have been designated in a previous report [15].

^e^Nucleotides comprising the end of the 3'-LTR and TSD were deleted in the contig and thus, TSD sequence found at the 5'-flanking region was presented.

### Functional motifs and structural features of *PwRn1 *and *AgCRs*

TSD detected in all of the *CsRn1*-like elements were found to be 4 bp, although there seemed to be no specific or preferential nucleotide in selecting the insertional targets among copies of each element and among the *AgCR *types (Table 1). The short inverted repeats of 3 bp, found at both ends of LTR in most retrotransposons (5'-TGT...ACA-3') [18], were slightly modified either to 5'-TGT...AAA-3' or to 5'-TGT...ATA-3' (Additional file 1). The LTR pairs flanking each of the *AgCR *elements displayed >97% nucleotide identities, suggestive of their recent expansion in the mosquito genome [19]. The putative primer-binding site of these elements for the synthesis of the first cDNA strand (5'-TGGTGAG/CCCCGT/A-3') was complementary to the nucleotides at the 3'-end of bovine t-RNA^Trp^. An additional priming site (polypurine tract) for the synthesis of the second cDNA strand was also found in the direct upstream region of 3'-LTR.

The internal regions of these elements contained ORFs with different expression strategies for their autonomous retrointegration. *PwRn1 *had an ORF similar to that of *CsRn1*, in which the *gag *and *pol *genes were fused together in a single frame (Figure 1). Of the 14 *AgCR *elements analyzed, nine elements utilized two ORFs overlapped by -1 frameshifting to encode the Gag and Pol proteins, while the other six elements contained two ORFs with an identical reading frame, which were intervened by a short nucleotide sequence ranging from 60 to 165 bp (Figure 2). The amino acid sequences of Gag proteins in retrotransposons generally diverge very rapidly so that they display a low level of sequence identity to one another, except for the functional Cys-His signature (CX_2_CX_4_HX_4_C; CCHC) [20]. The proteins, however, showed a high degree of amino acid conservation over their entire lengths in these *CsRn1-*like retrotransposons. They had the unique CHCC signature (CX_2_HX_9_CX_3_C) in the C-terminal regions, while it was imperfect in 3 elements, *AgCR-5*, *-7 *and *-9*. The α-helix-rich secondary structures of the Gag proteins were similar to those of the other *Ty3/gypsy*-like LTR retrotransposons (Additional file 2). The enzymatic domains contained in the Pol proteins were also well conserved with the conventional motifs/signatures: DT/SG tripeptide of PR, F/YXDD tetrapeptide of RT, DAS tripeptide of RH, and HHCC and DDE signatures of IN (data not shown). A series of LTR retrotransposons have an additional motif in the C-terminal region of IN, which is implicated in non-specific binding to the target site and/or to LTR via the best-conserved GPY/F residues [21,22]. This motif could also be detected in the corresponding proteins of *PwRn1 *and *AgCR*s with the exceptions of *AgCR-7 *and -*14*. Neither the chromatin organization modifier domain (chromodomain) nor ENV-like protein was detected in these elements.

### Phylogenetic analysis of *CsRn1*-like retrotransposons

**Figure 3:**
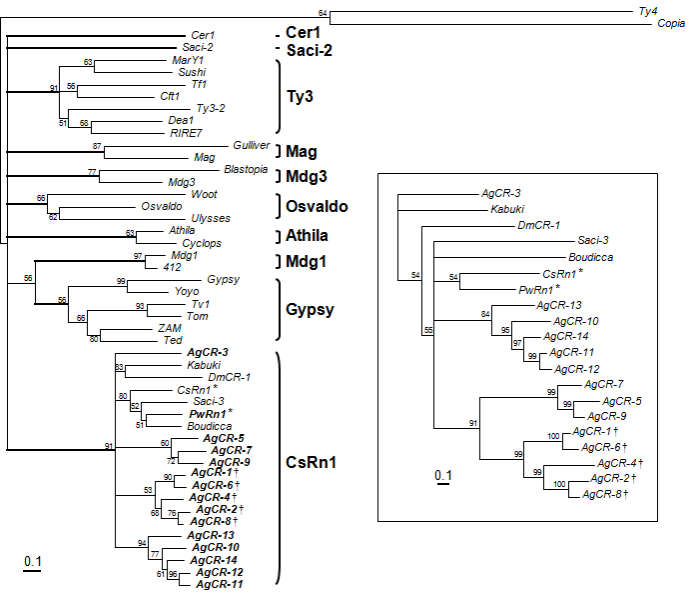
**Phylogenetic relationships between the *CsRn1*-clade members and the other *Ty3/gypsy*-like retrotransposons**. The analysis was performed with a concatenate of reverse transcriptase, RNase H, and Zn finger and DDE domains of integrase, using the maximum likelihood method of TREE_PUZZLE. The tree was rooted with *Ty4 *and *Copia*. Quartet supporting values are presented at each of the branching points. Elements characterized in this study are distinguished by the boldface letters. The symbols (*, †) found at the ends of element names indicate those with a single and two separated open reading frames, respectively. The tree in box was similarly obtained with the Gag sequences of *CsRn1*-like elements.

We obtained an alignment of Pol proteins from a total of 48 elements including the 15 elements isolated in this study. Amino acid positions for the three functional domains (RT, RH and IN; 510 aa) were concatenated and used in a phylogenetic analysis with the quartet-puzzling maximum likelihood method. The representative elements belonging to the previous eight clades of *Ty3/gypsy*-like LTR retrotransposons [22], as well as those of *CsRn1 *clade [9,12], were selected for the analysis. As shown in Figure 3, each of the sequences was well separated into the corresponding clades with significant quartet puzzling frequencies. The *PwRn1 *and *AgCR*s were categorized into the tightly conserved *CsRn1 *clade with a high level of quartet puzzling support (91%), as was predicted by the similarities in the primary structures of the Gag and Pol proteins. *PwRn1 *formed a subclade with its trematode neighbors (supporting value, 80%). The *AgCR *elements were further segregated into four distinct clusters and those with separated ORFs were monophyletically distributed in the tree, even though the supporting value was relatively low (53%; the elements were marked with †). *Saci-2 *of *S. mansoni*, which encodes an unusual Gag protein with the CCCH motif [12], occupied a unique phylogenetic position. Another tree based on the Gag sequences of *CsRn1*-like elements showed a topology similar to that of the Pol tree (boxed tree in Figure 3), while the phylogenetic relationships especially among the *Anopheles *elements with different expression strategies could be resolved more evidently with the rapidly evolving Gag sequences. The tree proposed that *Anopheles *elements with the separated ORFs have evolved from an ancient form(s) with two overlapped ORFs (91% support).

### Genomic distribution and mobile potential of *PwRn1*

**Figure 4:**
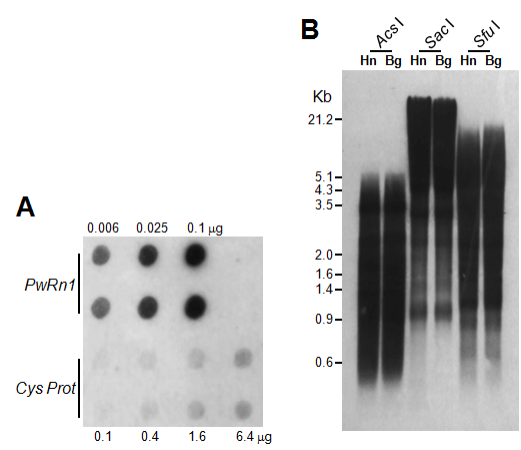
**Copy number and genomic distribution of *PwRn1***. (A) Reverse dot-blot analysis. The membrane was dotted in duplicate with varying amounts of Pw-D-13 fragment as shown on the top and probed with the genomic DNA of diploids. The blots of cysteine protease (Cys Prot) were used as a single copy control. The signal intensities were compared to estimate the copy number of *PwRn1*. (B) Southern blotting of *PwRn1 *with the genomic DNAs of diploid (Haenam, Hn) and triploid (Bogil-do, Bg) *Paragonimus westermani*. Restriction endonucleases used for the digestion of DNAs are presented at the top. The positions of DNA size standards (in kb) are shown on the left.

**Figure 5:**
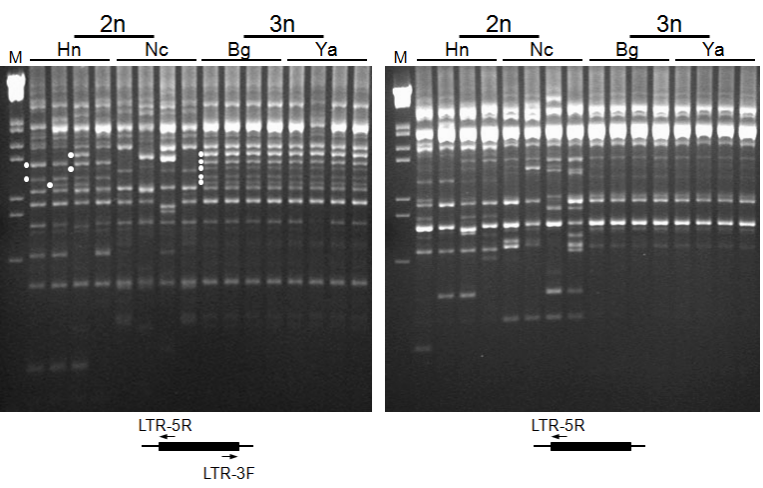
**Banding patterns of inter-retrotransposon amplified polymorphism (IRAP)**. Genomic DNAs were separately extracted from individuals of *P. westermani *(diploids from Haenam, Hn and Nanchang, Nc; triploids from Bogil-do, Bg and Youngam, Ya). The DNAs were used in PCR with the *PwRn1 *LTR-specific primers, as shown at the bottom. The amplified fragments were electrophoresed on agarose gels and visualized by ethidium bromide staining. White circles indicate triploid genome-specific IRAP markers, which were shared with the Korean diploids. M, lambda DNA/*Eco*R I + *Hin*d III marker.

**Figure 6:**
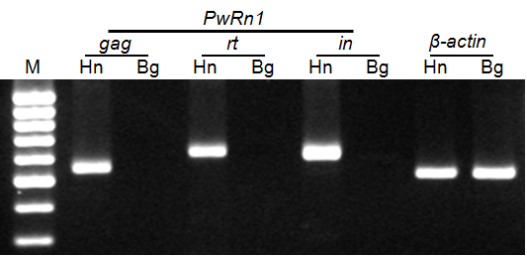
**Amplification of *PwRn1 *transcripts**. Reverse-transcription PCR (RT-PCR) was performed with the total RNAs extracted from diploid (Haenam, Hn) and triploid (Bogil-do, Bg) worms and *PwRn1*-specific primers. The RNAs were examined by conventional PCR to verify the absence of any contaminating genomic DNAs, prior to the RT-PCR (data not shown). The PCR products were analyzed by agarose gel-electrophoresis. A primer pair for the β-actin gene was used as a house-keeping gene control. M, 100-bp DNA ladder.

The copy number of *PwRn1 *was estimated over 1,000 per haploid *Paragonimus *genome via dot blotting (Figure 4A). A Southern blot analysis showed that these *PwRn1 *copies are interspersed throughout the genome, rather than being tandemly arrayed or being clustered at limited loci (Figure 4B). However, the blotting could not distinguish the distribution patterns of *PwRn1 *between diploid and triploid genomes, mainly due to the high copy number and resulting smeared hybridization signals. For a more detailed comparison of *PwRn1*-occupied loci, IRAP analyses were conducted with the individual genomic DNAs and *PwRn1 *LTR-specific primers (Figure 5). Numerous bands were amplified from genomic loci intervened between two copies of *PwRn1*. The patterns were found to be homogeneous among the triploid individuals and almost all of the triploid markers were detected in at least one of the diploid genomes, especially with the Korean origin (see the bands marked with white circles in Figure 5). Conversely, polymorphic markers were substantially observed among the diploid genomes at intra- and interpopulation levels. Analyses of more parasite individuals (up to 15) showed similar results (data not shown). Given the fact that the sporadic replication of an active retrotransposon introduces intergenomic polymorphism [23], this observation may reflect a differentially preserved mobile potential of *PwRn1 *depending on the karyotype of its host genome; the element has maintained the ability to produce progeny copies in diploids, whereas it has ceased to retrointegrate into new genomic loci in the triploid populations. In accordance with this suggestion, mRNA transcripts of the element were detected in a diploid population, but not in a triploid one (Figure 6). The intra-genomic expansion of retrotransposons initiates by transcribing their mRNAs using host RNA polymerase. By adapting to this unique replication mode, host defense mechanisms have diversely evolved by focusing on the protection of transcription and/or destruction of mRNA transcripts, including chromatin modification and RNA interference. Therefore, *PwRn1 *was likely to be suppressed at the initial transcription stage in the triploid population, although no relevant mechanism could be addressed in this study.

## Discussion

Since the first detection of LTR retrotransposons with an unusual capsid protein in a trematode and insect species [9,24], a series of similar elements have been isolated from genomes of the other trematodes and insects. They share unique properties such as PBS for t-RNA^Trp^, 4 bp of TSD and tight amino acid conservation in the encoded proteins. The most conspicuous feature, nevertheless, can be found in the amino acid sequences of Gag proteins. In general, the first ORF contained in various retrotransposons diverge rapidly so that they display low levels of sequence identity to one another, except for the active Cys-His motif (CX_2_CX_4_HX_4_C) [20]. However, the proteins of *CsRn1*-like elements have been known to conserve a novel sequence motif of CX_2_HX_9_CX_3_C (CHCC), instead of the conventional CCHC motif. These facts have suggested that members of this clade are originated from an intermediate form of reverse-transcribing elements, which have emerged in the common ancestor of the monophylyl ecdysozoan and lophotrochozoan animals. Retrotransposons characterized from the lung fluke and mosquito possessed all of these features and thus, further supported the notion that these elements are selectively expanded in the lower animal taxa.

The Gag protein of retrotransposons functions as a nucleocapsid protein during the assembly of virus-like particle (VLP), which is thought to be an important intermediate in retrotransposition [25]. The origin and functional relevance of Gag found in the *CsRn1*-like elements could not be properly addressed, because neither cellular nor the other retrotransposon-related protein was detected during homolog searches based on the BLAST algorithm and Hidden Markov models. Considering the well-conserved, α-helix-rich secondary structure (Additional file 2) and the active replication of these elements including *CsRn1 *[26], the proteins seem to have nucleic acid-binding capacity to form VLPs. Malik and Eickbush [7] have proposed the chimeric origin of LTR retrotransposons and retroviruses, between a preexisting element and non-LTR element/host gene. This may reflect the genetic flexibility endogenous in the mobile elements to generate novel functional/structural variants. A *gag *gene equipped in the preexisting element is likely to have been substituted by a presently unidentified host gene with the unusual motif during the evolutionary start point of *CsRn1*-like elements.

Different expression strategies were observed among the *CsRn1*-like elements (Figure 1 and 2; see also [15]). A single primary transcript is responsible for the production of multiple retrotransposon proteins, which are essential for the autonomous replication of the corresponding element, and is subjected to ribosomal frameshifting to maintain a proper ratio between the Gag and Pol proteins [27]. Genetic events including insertion/deletion of single nucleotide around the frameshifting region are occasionally attributable to the generation of retrotransposons with a single fused ORF. The Gag and Pol proteins are produced by proteolytic cleavage of a single translation product in the cases of single-ORF variants [28,29]. The variant form will replace the previous one in the host genome, via the highly efficient VLP formation [29]. The structural variation was not detected with the multiple *PwRn1 *copies retrieved by PCR. The ancient copies with two overlapped ORFs might have decayed in the *Paragonimus *genome, mainly due to the low propagation rate. Otherwise, diagnostic base substitutions in priming regions of the variant copies might lead to a failure in the amplification of the corresponding DNA segments.

Retrotransposons generally show very low retrotransposition and excision rates, and their expansion is largely restricted to germ cells and/or an early developmental stage [18]. These elements, however, become unstable under specific circumstances and can rapidly expand in the host genomes by increasing their replication frequency. The mobile potential of *PwRn1 *seemed to be differentially preserved among *P. westermani *populations; the element maintained its activity in diploids, while a majority of the activity had been suppressed in triploid individuals (Figures 5 and 6). In association with the origin of the triploid worms, an allopolyploidic hybridization occurred in ancestral diploids at the intra- or interspecies level has been suggested as a relevant genomic event ([13] and references therein). IRAP markers shared in the diploid and triploid worms are likely to represent genomic loci flanked by *PwRn1 *copies in the putative ancestral diploids, and may provide further genomic evidence supporting the proposed mechanism. Together with biotic/abiotic stresses such as aridity [30], polyploidization can boost the mobile potential of various retrotransposons [31,32]. Therefore, it was unexpected to find that *PwRn1 *is less active in triploid genomes. Host genome surveillance for retrotransposons is likely to be reinforced in association with an increase in genomic dosage [33]. Alternatively, *PwRn1 *itself would give a feedback effect as a mechanism to compensate for the amplified copy number, following the hybridization process. Phylogenetic and comparative analyses with the multiple *PwRn1 *copy sequences will be informative to elucidate the evolutionary status/mode of the element among the *Paragonimus *populations with different polyploidy.

There have been various reports on the comparison of *P. westermani *genomes to address the issue regarding the origin of polyploid genomes (reviewed in [13]). Molecular data such as the nucleotide sequences of mitochondrial and ribosomal DNAs dichotomized the genomes into the northeast (China, Japan, Korea and Taiwan) and south (the Philippines, Thailand and Malaysia) Asian groups, but failed in establishing more detailed relationships among the northeast populations with different polyploidy [34]. The retrointegration of retrotransposons is an irreversible event and the integration site is randomly selected [35,36]. These characteristics of mobile elements are particularly suitable for the comparative population studies at the intra- and interspecies levels [37,38]. In this study, we provided molecular evidence demonstrating genomic conservation and/or diversification between Korean diploids and triploids by analyzing intergenomic distributions of *PwRn1 *(Figure 5). The data suggests that IRAP and locus-specific typing of active retrotransposons can be an informative genetic marker in exploring the flow of haplotypes among *P. westermani *populations, and in elucidating the controversial hypotheses on the origin of the triploid populations. In plants, transposable elements are believed to be reactivated very early following polyploid formation and thus contribute to genetic diversity in the polyploidy species [41]. However, understanding on the role of these elements in polyploidy evolution is just beginning to emerge. The polyploid genomes are less common in animals than in plants. Our results on the suppressed mobile activity of *PwRn1 *in the triploid *Paragonimus *genomes can boost research to examine the evolutionary impacts of retrotransposons in polyploid animal genomes.

## Conclusion

With the completion of genome projects from numerous eukaryotic organisms, comprehensive studies on the mobile genetic elements have been conducted with the whole genomic sequences. These studies have broadened our knowledge on the dynamics and impacts of retrotransposons in the host genomes. Despite the rapid accumulation of data, however, only limited information is currently available from a few species in the wide phylum Platyhelminthes. It is apparent that these invertebrate animal genomes contain diverse retrotransposons and that these elements are actively involved in the remodeling and diversification of their host genomes. Our results on the *CsRn1*-like elements of *P. westermani *and *A. gambiae*, especially in association with the diversified expression strategies, can make a significant step toward a better understanding of evolutionary episode within the unique *CsRn1 *clade of LTR retrotransposons, which is specifically expanded in the lower animal taxa. The differentially preserved mobile potential of *PwRn1 *and resulting genomic polymorphism in diploid individuals will also be helpful in designing a comparative genomic study with the northeast Asian populations of *P. westermani*.

## Methods

### Parasite

Experimental dogs were orally challenged with the metacercariae of *P. westermani*, which were collected from naturally infected crayfish in endemic areas of Korea (Haenam, 2n; Bogil-do and Youngam, 3n) and China (Nanchang, Jiangxi Province, 2n) [39]. Five months after the infections, adult worms of the parasite were harvested from the dogs' lungs. The worms were washed five times with physiological saline at 4°C and used for the extraction of DNA and RNA with the Wizard DNA Purification Kit (Promega, Madison, WI, USA) and TriZol reagents (Invitrogen, Carlsbad, CA, USA), according to the manufacturers' instructions. Materials from diploid worms (Haenam, Korea) were commonly used in this study; otherwise the sources were specifically indicated. The use of animals was approved by the Animal Ethics Committee of Korea Food and Drug Administration (protocol number NIH-05-09).

### Isolation of a *CsRn1*-like retrotransposon from *P. westermani*

The retrotransposon-related sequences were retrieved via a degenerate PCR method from the *P. westermani *genome, as described previously [14]. Of the sequences obtained, a clone (Pw-D-13), that showed a significant degree of sequence identity with *CsRn1 *was selected for further characterization. Genomic DNA library of the parasite constructed using the lambda FIX II vector system (Stratagene, La Jolla, CA. USA) was screened with the DNA probe according to the standard procedure of plaque-lift hybridization. The inserts of two positive clones were amplified by long-range PCR using primers designed from the vector regions (5'-CTAATACGACTCACTATAGGGCGTCG-3' and 5'-CCCTCACTAAAGGGAGTCGACTCG-3') and LA *Taq *polymerase, under the standard cycle condition (Takara, Shiga, Japan). The amplified products were digested with *Kpn *I and *Xho *I, and were cloned into pBluescript II SK(-) phagemid (Stratagene) for sequencing. The nucleotide sequences were automatically determined with the ABI PRISM 377 DNA Sequencer (Applied Biosystems, Foster City, CA, USA) and the BigDye Terminator Cycle Sequencing Reaction Kit (Perkin Elmer Corporation, Foster City, CA, USA). To ensure the accuracy of the reactions, nucleotide sequences from both strands were determined. Contig sequences were obtained by overlapping the sequence information. These contigs were then compared with one another to determine the full-unit of a retrotransposon encompassing the Pw-D-13 clone, which was designated *P. westermani retrotransposon 1 *(*PwRn1*).

### *In silico *identification of insect retrotransposons homologous to *CsRn1*

Whole genomic sequences of A. gambiae and D. melanogaster deposited in the GenBank were surveyed by BLAST algorithms using the amino acid sequences of CsRn1 Gag protein, which showed a unique Cys motif [9], as a query. The hits showing amino acid positive values greater than 45% throughout the whole query sequence were subject to further analyses to determine the full-units of retrotransposons encompassing each of the gag genes. Multiple scaffold sequences were compared with one another and then, a common genetic element contained within them was isolated. The terminal repeats flanking a protein-encoding internal region were determined by analyzing the sequences with bl2seq at National Center for Biotechnology Information (NCBI, ) and further verified by recognizing the duplicated target sequences from the direct upstream and downstream regions of the elements. BLAST searches with the entire nucleotide sequences of the elements were followed to confirm their integrity and to retrieve additional copy sequences from the identical databases. The coding profiles and conserved protein domains were predicted using ORF Finder at NCBI and InterProScan programs at European Bioinformatics Institute (EBI, ), respectively. The homology patterns were also determined by a series of BLAST searches against the non-redundant genomic/protein databases of the GenBank.

### Southern and dot-blot hybridization of *PwRn1*

Genomic DNAs isolated from *P. westermani *adult worms (5 μg per each) were digested with restriction enzymes, *Acs *I, *Sac *I and *Sfu *I. After being separated on a 0.8% agarose gel, the restricted DNA fragments were transferred onto a nylon membrane (Hybond-N^+^; Amersham Pharmacia Biotech, Uppsala, Sweden) by capillary action in 10 × SSC. The blots were hybridized with the Pw-D-13 probe enzymatically labeled with the ECL Direct Labeling Kit (Amersham Pharmacia Biotech). The labeling, hybridizing, and signal detection conditions were determined under the manufacturer's instructions. For stringency washing, the membrane was washed twice in 0.1 × SSC containing 6 M urea and 0.4% SDS at 42°C for 20 min, and twice in 2 × SSC at room temperature for 5 min.

Various amounts of the Pw-D-13 DNA fragment were blotted onto a nylon membrane, according to the standard procedure of dot-blot hybridization. The membrane was hybridized with the genomic probe of *P. westermani*, which was prepared by sonicating the genomic DNA into small DNA fragments between 0.5 and 2 kb. For a single-copy control, a portion of the *Paragonimus *cysteine protease gene [GenBank:U70537], with a size of approximately 700 bp, was amplified from the parasite genome by PCR using a gene-specific primer pair (5'-TCAGTTGTCTTGTTGTCGTGG-3' and 5'-TGCCTGTTTCTCCTCATTCTTG-3') and blotted onto the membrane. Probe labeling and hybridizing conditions were identical to those for Southern blot hybridization. The intensities of signals were measured using the LAS-1000plus system (FUJIFILM, Tokyo, Japan) and compared with those of the control gene.

### Reverse-transcription PCR (RT-PCR)

Total RNAs were extracted from the diploid and triploid adult worms (10 worms per each) and treated with the RNase-free DNase (GIBCO BRL, Rockvile, MD, USA). Reverse transcription and following amplification of *PwRn1 *transcripts were carried out with the total RNAs and retrotransposon-specific primers, using RNA PCR Kit (AMV) (Ver. 2.1; Takara) under the manufacturer's instructions. The primers used were as follows: 5'-AGAGAGATGTGGCTACAGCG-3' and 5'-GTTTAAGTCGTGGACCTCAG-3' for *gag*; 5'-GCAGGTTGACACCAGACAAG-3' and 5'-ATCGGTTAACGGTCGGATAC-3' for *rt*; 5'-GTCGATGCAATCCGTTGGAC-3' and 5'-CCAGTGCACCCTACGACCTG-3' for *in*; 5'-GGCCATGTACGTTGCTATCC-3' and 5'-CAGAGAGAACAGTGTTGGCG-3' for β-actin gene. The absence of any contaminating DNA was confirmed by preparing reactions without reverse transcriptase during the first round of cDNA synthesis and the β-actin gene was selected for a house-keeping gene control. The reaction products were resolved by agarose gel electrophoresis.

### Inter-retrotransposon amplified polymorphism (IRAP)

Primers were designed to match the 5'- and 3'-ends of a consensus PwRn1 LTR sequence in the outward directions, respectively (LTR-5R, 5'-AGGCAGGCCAGTGAAATCT-3' and LTR-3F, 5'-CGACTTAGTGCAACGAGCAC-3'). Genomic DNAs were individually extracted from diploid and triploid worms. The IRAP PCR was performed with reaction mixtures containing 20 ng of the genomic DNA, 1 μM of each primer, 0.2 mM of each dNTP precursors, 1.6 mM of MgCl2, and 1.25 units of Taq polymerase (Takara) in 2 mM Tris-HCl buffer (pH 8.0). PCR cycling parameters were as follows: 4 min at 94°C; 35 cycles of 40 sec at 96°C, 40 sec at 56°C, and 2 min at 72°C; 10 min at 72°C. The PCR products were analyzed by electrophoresis on 2% agarose gels (NuSieve 3:1 agarose; Cambrex Bio Science, Rockland, ME, USA) and visualized by staining with ethidium bromide.

### Phylogenetic analysis

Pol protein sequences of LTR retrotransposons were aligned with ClustalX and optimized with GeneDoc programs. The regions corresponding to each of the RT, RH and IN domain sequences were selected from the alignment, in order to increase the analytical resolution, compared to that obtained by using RT sequences alone [22,40]. The resulting concatenates comprising approximately 510 amino acid positions were adopted in a phylogenetic analysis for the construction of maximum likelihood tree using the quartet method implemented in TREE-PUZZLE (Ver. 5.2). The analytical options were as follows: JTT model for substitution, estimation of invariant site proportion from the input data, 1,000 puzzling steps assuming rate heterogeneity with eight gamma categories (the gamma distribution parameter alpha was estimated from the data set). Indels between pairs of sequences were regarded as missing data. Tree construction was performed by the neighbor-joining method. Gag sequences of the *CsRn1*-clade members were similarly examined by the phylogenetic approach. The phylogenetic trees were displayed by the TreeView program.

## Abbreviations

ENV: envelope; IN: integrase; IRAP: inter-retrotransposon amplified polymorphism; LTR: long terminal repeat; ORF: open reading frame; PR: protease; RH: RNase H; RT: reverse transcriptase; TSD: target site duplication; VLP: virus-like particle.

## Authors' contributions

YAB contributed to the experimental design, sequence analyses, sequence alignments and phylogenetic analyses, and drafted the manuscript. JSA performed experiments regarding construction and screening of the *P. westermani *genomic DNA library. SHK carried out the Southern and dot-blot hybridizations, and IRAP analysis. MGR participated in experimental design and bioinformatic analysis of the sequence data. YK helped to collect the experimental materials and to design the project. SYC oversaw the research project, contributed in its design and participated in editing the manuscript. All authors read and approved the final manuscript.

## Supplementary Material

Additional File 1 **Primer-binding site (PBS) and polypurine tract (PPT) conserved in the nucleotide sequences of *PwRn1 *and *AgCR*s**. Nucleotides in both termini of LTRs, PBS and PPT were compared among these *CsRn1*-like elements. The 3'-end of bovine tRNA^Trp^, complement to the putative PBS, is also presented at the top. Dots were introduced into the sequences to increase their homology values. Breaks marked with a double slash indicate the regions which were removed to shorten the alignment. Click here for file

Additional File 2 **Comparison of Gag sequences encoded by the *CsRn1*-clade members**. The amino acid sequences were aligned with ClustalX and optimized with GeneDoc. The shading pattern indicates difference in amino acid conservation for the individual positions and sequence identities are highlighted in black. The functional signature (CHCC) conserved in the *CsRn1*-like retrotransposons is marked with filled arrowheads. The filled cylinders at the top indicate regions corresponding to the α-helixes. Click here for file
