# Supplementary figures and images for: PwRn1, a novel Ty3/gypsy-like retrotransposon of Paragonimus westermani: molecular characters and its differentially preserved mobile potential according to host chromosomal polyploidy
Source: BMC Genomics. 2008 Oct 14;9:482. doi: 10.1186/1471-2164-9-482 (PMC2582038; doi:10.1186/1471-2164-9-482)

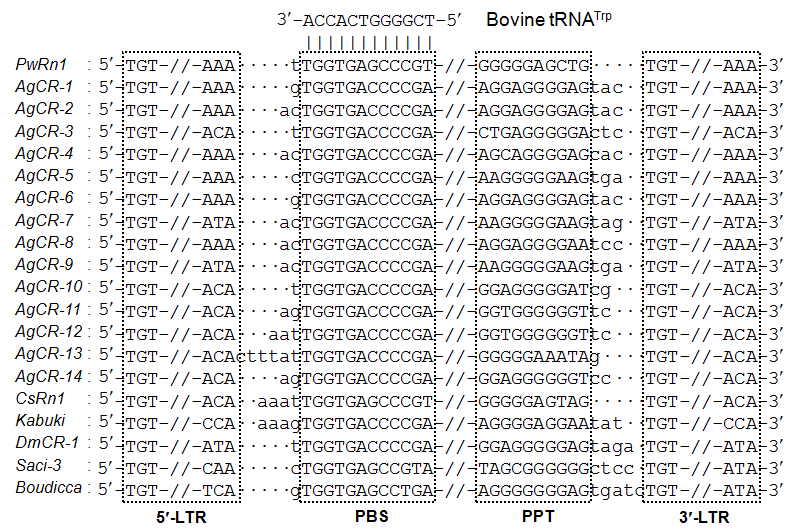

Supplement: Additional File 1 — Primer-binding site (PBS) and polypurine tract (PPT) conserved in the nucleotide sequences of PwRn1 and AgCRs. Nucleotides in both termini of LTRs, PBS and PPT were compared among these CsRn1-like elements. The 3'-end of bovine tRNATrp, complement to the putative PBS, is also presented at the top. Dots were introduced into the sequences to increase their homology values. Breaks marked with a double slash indicate the regions which were removed to shorten the alignment. [file 1471-2164-9-482-S1.tiff]

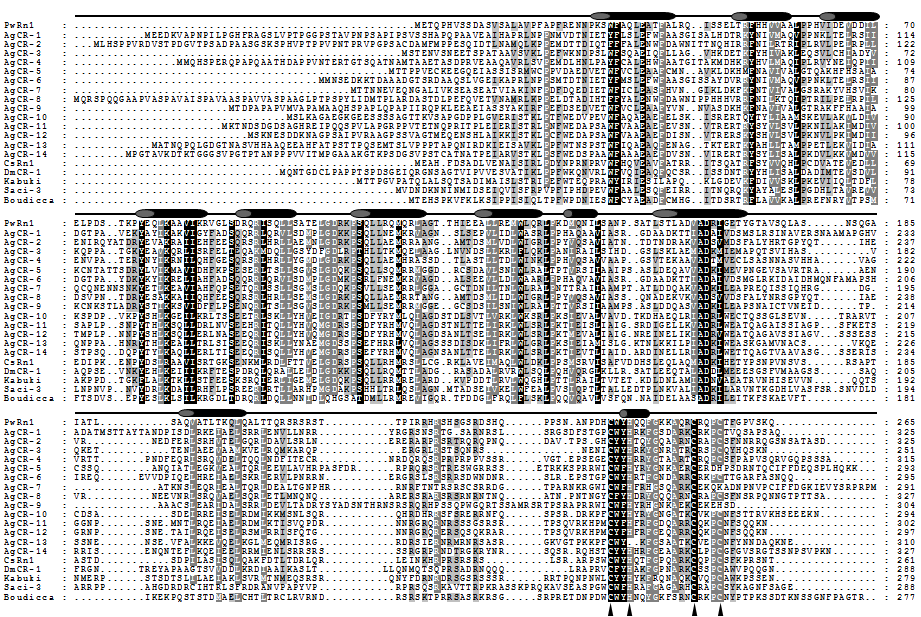

Supplement: Additional File 2 — Comparison of Gag sequences encoded by the CsRn1-clade members. The amino acid sequences were aligned with ClustalX and optimized with GeneDoc. The shading pattern indicates difference in amino acid conservation for the individual positions and sequence identities are highlighted in black. The functional signature (CHCC) conserved in the CsRn1-like retrotransposons is marked with filled arrowheads. The filled cylinders at the top indicate regions corresponding to the α-helixes. [file 1471-2164-9-482-S2.tiff]
